# Isolated Superior Mesenteric Artery Dissection: An Unusual Etiology of Epigastric Pain

**DOI:** 10.7759/cureus.25683

**Published:** 2022-06-06

**Authors:** Adedoyin Olawoye, Htin Kyaw, Ifeanyi F Nwosu, Cece E Ibeson, Tania Miah, Benjamin Weindorf, Thai Donenfeld, Arjun Basnet, Oladapo Adaramola, Geraldine C Nsofor, Abiola A Adebayo

**Affiliations:** 1 Internal Medicine, Maimonides Medical Center, New York City, USA; 2 Radiology, Maimonides Medical Center, New York City, USA; 3 Internal Medicine, Milton Keynes University Hospital, Milton Keynes, GBR; 4 Internal Medicine, Charing Cross Hospital Imperial College NHS Trust, London, GBR

**Keywords:** ultrasound scan, computed tomography, superior mesenteric artery dissection, visceral artery dissection, abdominal pain

## Abstract

Abdominal pain is a very common presentation in the emergency department (ED). The pain is often well-characterized and leads to the diagnosis, but often, the presentation is vague and nonspecific. Superior mesenteric artery (SMA) dissection is a rare cause of abdominal pain that presents with nonspecific epigastric pain and is common in males, middle age, and patients of Asian descent. A high index of suspicion is usually helpful with imaging modalities such as computer tomography (CT) scan and ultrasonography in experienced hands. A prompt diagnosis is vital to managing this disease which may range from non-surgical intervention with supportive therapy to invasive endovascular procedures and surgery. Here, we report a case of an isolated SMA dissection presenting with vague abdominal symptoms and highlight the need to explore the vascular etiology of abdominal pain as their diagnosis is often difficult and may result in irreversible bowel injury when missed.

## Introduction

A myriad of pathologies can present with abdominal pain, and one-third of the patients seen in the ED are diagnosed with nonspecific abdominal pain [1]. The pain of vascular etiology is less considered during the initial evaluation. Abdominal pain of vascular origin presents a diagnostic challenge as the pain is typically nonspecific and lacks the physical signs and preliminary laboratory and radiological findings expected for the severity of the pain [2]. A diagnostic delay may lead to catastrophic and often irreversible bowel injury. Splanchnic vascular dissection and mesenteric artery thrombosis are some of the vascular causes of acute abdominal pain. A splanchnic vascular dissection can cause abdominal pain either by the shearing force of the dissection on the vessels or the distal ischemia to the structure supplied by the blood vessel [3,4].

Spontaneous visceral artery dissection is rare [5]. Isolated spontaneous superior mesenteric artery (SMA) dissection represents the most common form of visceral artery dissection, although most SMA dissection occurs as an extension of an abdominal aortic dissection [3,6]. SMA dissection should be considered a differential diagnosis in patients with risk factors presenting with a vague abdominal pain and characteristics that do not fit into any typical intrabdominal pathology [6].

## Case presentation

**Figure 1:**
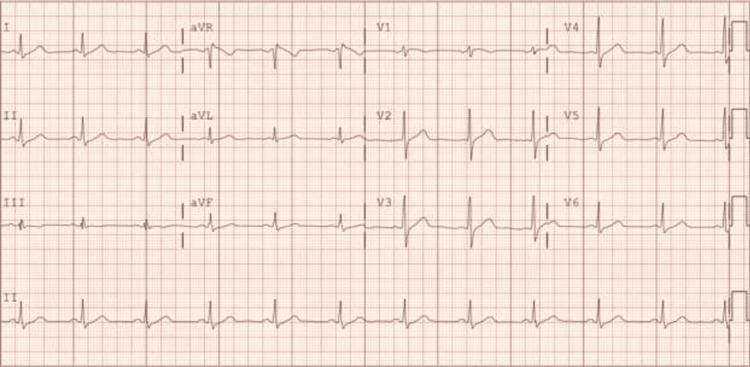
EKG showing sinus rhythm

A 54-year-old Asian male with a past medical history of hepatitis B infection on tenofovir, gastroesophageal reflux disease, cholelithiasis post laparoscopic cholecystectomy presented with 3 days of mild epigastric non-radiating abdominal pain. The pain was unrelated to food but was worse with movement with no relieving factors. He had one episode of non-bilious, non-bloody vomiting. The patient denies change in bowel habits, fever, chills, bloody stools, dizziness, jaundice, rash, or chest pain. Current smoker, with a 35-pack-year history. On examination, he was not in acute distress, afebrile, not icteric, and not dehydrated. An abdominal exam revealed a soft, mildly distended abdomen with mild diffuse tenderness to palpation. Laboratory results were normal other than aspartate transaminase and alanine transaminase which were mildly elevated to 116 (10-33 iu/l) and 94 (6-47 iu/l), respectively. A venous gas lactic acid level was 1.5 (0.5-1.6 mmol/l). The electrocardiogram (EKG) showed sinus rhythm (Figure 1).

**Figure 2:**
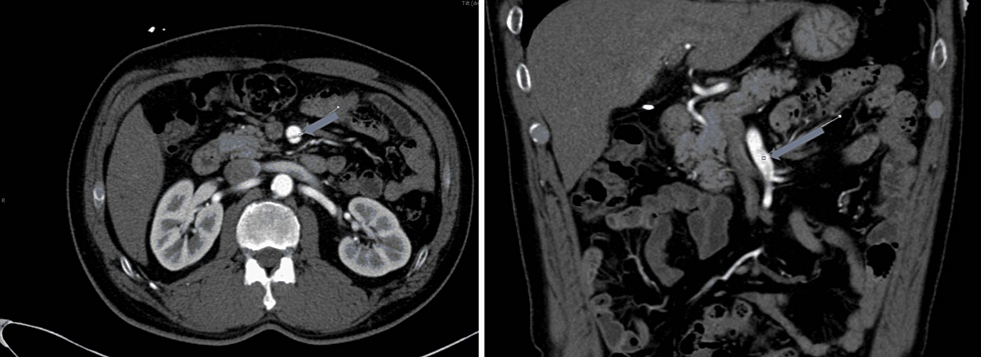
CT angiogram of abdomen and pelvis showing an intimal tear of the SMA SMA: superior mesenteric artery

A bedside abdominal sonogram to evaluate gall bladder disease and the abdominal aorta suspected an SMA aneurysm. A CT angiogram abdomen and pelvis confirmed an isolated SMA dissection with no flow limitations and no abdominal aortic aneurysm (Figure 2).

The patient was managed conservatively with intravenous fluid hydration, bowel rest, pain control, and low-dose aspirin. A follow-up CT angiogram a month after discharge showed stable disease.

## Discussion

Common risk factors for arterial dissections include hypertension, atherosclerosis, and connective tissue diseases, but these play a limited role in the pathogenesis of SMA dissection [7,8]. Although our patient was not hypertensive, he had some known risk factors for SMA dissection. Risk factors include male gender, hypertension, smoking, SMA-distal aorta angle, and Asian ethnicity [9,10]. CT angiography of the abdomen is the imaging of choice to diagnose SMA dissection. Still, when performed by experienced hands, a preliminary abdominal ultrasound may be a good alternative, as shown in this patient. In this case, the abdominal pain did not follow a specific pattern that could be attributed to vascular pathology. The patient does not have a history of atrial fibrillation, and with normal lactate, the suspicion of an ischemic bowel from acute mesenteric ischemia was low in the differential diagnosis consideration. Aortic dissection was a possible differential given the smoking history.

The management of isolated SMA dissection is mainly conservative with intravenous hydration, anticoagulation with heparin, and surgical options reserved for patients with worsening symptoms, progression of dissection, and bowel ischemia [11,12]. Of the surgical options, endovascular treatment such as balloon angioplasty and stent placement are mostly employed in uncomplicated cases of SMA dissection [13]. In patients with arterial rupture or bowel necrosis, endoaneurysmorrhaphy, patch angioplasty, thrombectomy, and bypass grafting are options for management [13]. The role of antiplatelet remains uncertain, and studies have shown no benefits with antithrombotic treatment [7]. Although this patient had a benign course and required only medical therapy, the disease course would have been different if he had a more severe dissection, bowel ischemia, necrosis, or a delay in diagnosis due to his nonspecific symptomatology. Therefore, a high index of suspicion for visceral vascular disease is necessary when evaluating a patient with atypical abdominal pain that does not fit into the pattern for common abdominal conditions, and even more with an initial negative result for common pathologies.

## Conclusions

Visceral artery dissection can present with acute abdominal pain, of which SMA dissection is the most common cause. Investigations directed toward assessing vascular pathologies should be considered early when the cause of abdominal pain is uncertain, especially in patients with existing risk factors.
